# Investigation of the Effectiveness of Mindfulness-Based Yoga Training in Individuals with Fibromyalgia: A Randomized Controlled Trial

**DOI:** 10.3390/healthcare14121792

**Published:** 2026-06-21

**Authors:** Ebru Durusoy, Abdülhakim İbrahim Ulusoy, Özge Önürmen Zeyrek, Sebahat Yaprak Çetin, Sevil Bilgin, Edibe Ünal

**Affiliations:** 1 Department of Physiotherapy and Rehabilitation, Institute of Health Sciences, Hacettepe University, Ankara 06800, Türkiye; 2 Department of Physiotherapy and Rehabilitation, Faculty of Health Sciences, Istanbul Gelisim University, İstanbul 34310, Türkiye; 3 Avcılar Murat Kölük State Hospital, İstanbul 34320, Türkiye; ulusoydr@gmail.com; 4 Department of Physical Medicine and Rehabilitation, Sakarya Training and Research Hospital, Sakarya 54100, Türkiye; ozgeonurmen@gmail.com; 5 Department of Physiotherapy and Rehabilitation, Faculty of Health Sciences, Akdeniz University, Antalya 07058, Türkiye; fzt.ycetin@gmail.com; 6 Department of Physiotherapy and Rehabilitation, Faculty of Physical Therapy and Rehabilitation, Hacettepe University, Ankara 06800, Türkiye; sevil.bilgin@hacettepe.edu.tr (S.B.); edibeunal@gmail.com (E.Ü.)

**Keywords:** fibromyalgia, mindfulness, yoga, telerehabilitation

## Abstract

**Background:** Fibromyalgia is a chronic condition characterised by widespread pain, fatigue, sleep disturbances, and psychological symptoms. Mindfulness-based approaches are increasingly used as complementary interventions for symptom management. This study aimed to investigate the effectiveness of mindfulness-based yoga (MBY) delivered via telerehabilitation in individuals with fibromyalgia. **Methods:** This trial included 64 women with fibromyalgia who were randomly assigned to an 8-week mindfulness-based yoga program delivered via telerehabilitation or active control group including walking and physiotherapy modalities. Both groups received patient education at the outset. Assessments were conducted before and after the intervention. Outcome measures included fatigue, anxiety, depression, sleep quality, symptoms associated with central sensitization, kinesiophobia, pain intensity, mindfulness level, impact of fibromyalgia on life, biopsychosocial status, and pain catastrophising. Data were analyzed using mixed-design analysis of variance (ANOVA), with additional *t*-tests and analysis of covariance (ANCOVA) conducted for post hoc analyses. **Results:** Compared to the control group, the mindfulness-based yoga (MBY) group showed more pronounced improvements in terms of fatigue, anxiety, symptoms associated with central sensitization, biopsychosocial status, symptom severity, catastrophising about pain, ruminative thoughts about pain, and cognitive dimensions of pain. Although no significant differences were found between groups for other variables, intra-group improvements were observed in the MBY group. **Conclusions:** It was concluded that the MBY intervention administered via telerehabilitation is a viable complementary approach to traditional treatments in reducing the symptom burden of fibromyalgia. It was thought that the effectiveness of the research could be increased by conducting studies involving long-term follow-up assessments and investigating the integration of different mindfulness-based telerehabilitation interventions into the clinical setting.

## 1. Introduction

Fibromyalgia (FM) is a chronic and multidimensional condition characterised by widespread chronic pain, fatigue, sleep disturbances, cognitive difficulties, and mood changes [1]. *t* is currently defined as a nociceptive pain disorder associated with central sensitisation mechanisms and is recognised as a complex clinical picture resulting from the interaction of biopsychosocial factors [2]. However, although its aetiology and pathogenesis are not yet fully understood [3], it is most commonly seen in women aged 30–35 and is approximately three times more prevalent in women than in men [4]. The disease significantly reduces individuals’ functional capacity and quality of life. The primary goal of treatment is to reduce symptoms and develop coping skills so that patients can effectively maintain their daily lives. In this regard, pharmacological and non-pharmacological approaches must be applied together [5]. Exercise approaches, which are non-pharmacological methods, are strongly recommended by the European League Against Rheumatism (EULAR) for the treatment of fibromyalgia due to their positive effects on pain, physical function and general well-being, ease of application, low cost and safety [6]. Exercises defined as mind–body exercises, such as yoga, tai chi, and qi gong, are holistic approaches aimed at enhancing mind–body interaction. Although previous studies have suggested potential benefits of these approaches for reducing FM symptoms and improving quality of life, the strength and consistency of the evidence remain limited [7,8].

MBY is a mind–body exercise that integrates the physical and mental components of hatha yoga with the fundamental principles of mindfulness meditation. The practice consists of postures, breathing techniques, and mindfulness exercises. This approach aims to direct the individual’s attention consciously to the present moment and to notice thoughts, feelings, and bodily sensations without judgement [9,10]. A limited number of studies on MBY in individuals with FM have reported improvements in pain, fatigue, mood, functionality, pain catastrophizing, acceptance, and adaptive coping strategies [11,12,13,14]. However, the current evidence remains limited by small sample sizes, heterogeneous intervention protocols, differences in control conditions, limited follow-up periods, and methodological variability. In addition, the face-to-face delivery of guided mind–body practices such as yoga, Pilates, tai chi, and qigong may be limited by space requirements, time constraints, cost, difficulties in participation, and challenges in maintaining treatment continuity [15,16,17]. At this point, telerehabilitation applications are emerging as an important alternative, facilitating communication between patients and healthcare professionals, thereby increasing access to healthcare services and enabling the provision of long-term and sustainable services [18,19]. Telerehabilitation is a widely used and cost-effective method for managing neurological, cardiorespiratory, and musculoskeletal disorders [15]. This approach is also applied in FM management and has been reported to have positive outcomes on symptom burden, pain intensity, depression, pain catastrophising and quality of life [20,21]. However, research on telerehabilitation-based yoga for FM is quite limited in the literature, and most of the existing studies focus primarily on body awareness-based approaches [15].

Furthermore, although the existing literature reports promising results for MBY interventions, the majority of studies consist of small-sample pilot studies, and studies involving randomisation are rarely encountered. Most studies are compared with passive or waiting list control groups [11,12]. On the other hand, no randomised controlled trial (RCT) has been found in the literature where the guided components of MBY specific to FM are applied via telerehabilitation. Furthermore, the absence of studies comparing standard treatment approaches with current clinical guidelines constitutes a significant methodological gap. This RCT was designed to evaluate the effectiveness of MBY training delivered via telerehabilitation in individuals with FM.

## 2. Methods

### 2.1. Study Design and Participants

This study is a two-group, parallel-design RCT. Individuals in the control group received patient education, traditional physiotherapy modalities, and a recommendation to walk three days a week. The MBY group, designed as the study group, received patient education and mindful yoga training via telerehabilitation. The MBY group participated in one online guided session per week (led by a specialist physiotherapist with a mindful yoga instructor certification). On two days per week, they continued their home practice using the video or audio recordings provided to them. The research was conducted at a single centre, a secondary state hospital, and was carried out after obtaining approval from the Istanbul Gelişim University Rectorate Ethics Committee (Decision No: 2023-07-129) and the Istanbul Provincial Health Directorate (approval dated 13 March 2024 and numbered 2024/05). The study was retrospectively registered at ClinicalTrials.gov under the identifier NCT07439900 on 26 February 2026, after the initiation of participant recruitment. The delay in registration was due to administrative and procedural reasons encountered during the registration process. However, ethical approval and institutional permissions had been obtained prior to participant recruitment, and the predefined study protocol, including eligibility criteria, intervention procedures, outcome measures, and the statistical analysis plan, remained unchanged throughout the study. All participants were informed and signed a written consent form before being included in the study.

### 2.2. Eligibility Criteria

The sample for this study consisted of volunteers who had been diagnosed with fibromyalgia and who had applied to the relevant state hospital. Participants included individuals whose native language was Turkish, who were literate, aged 18 years or older, had been diagnosed with fibromyalgia, had stable medical treatment for the past three months, were not expected to undergo medication changes during the study period, and had a follow-up appointment scheduled for three months later. Participants were also screened for access to a computer, tablet, or smartphone and an active internet connection; the ability to use digital devices and the internet to participate in video conference sessions, or having a relative who could provide support in this regard, was accepted as one of the inclusion criteria. Those with uncontrolled or clinically significant additional diseases (chronic obstructive pulmonary disease, congestive heart failure, endocrine system diseases, neurological or psychiatric diseases, etc.), those with a history of malignancy, those with functional loss due to previous surgery on the spine or extremities, pregnant women, those continuing another rehabilitation programme, those with any condition preventing exercise, and individuals who declined to participate in the study were excluded.

### 2.3. Recruitment

Participants were recruited from the Physical Therapy and Rehabilitation outpatient clinic of a second-tier state hospital. Participant recruitment was conducted between 14 March 2024 and 30 December 2024. Individuals who were diagnosed with FM or whose existing FM diagnosis was confirmed by a specialist physical medicine and rehabilitation physician according to the 2016 American College of Rheumatology (ACR) diagnostic criteria were assessed for eligibility for the study.

### 2.4. Sample Size Estimation

When determining the sample size, the results of previously conducted studies in the literature or the generally accepted ‘effect size’ in the relevant field may be used. The effect size was calculated using data reported in a previous randomized controlled trial investigating yoga-based intervention in individuals with fibromyalgia [12].

When calculating the sample size, the first type error margin (α) was taken as 0.05 and the test power (1-β) as 0.80. In the calculations, the mean (35.5) and standard deviation (17.6) values of the Post–Revised Fibromyalgia Impact Questionnaire (FIQR) total score variable of the ‘MBY’ group and the mean (48.7) and standard deviation (18.9) values of the Post–FIQR total score variable of the ‘Control’ group were used. Based on these data, the effect size was calculated as 0.72. The analyses revealed that a minimum of 32 participants in each group would be sufficient to detect a statistically significant difference at an 80% power level.

### 2.5. Randomization

The allocation of participants to groups was carried out using a simple randomisation method. During the randomisation process, each individual drew lots to determine their assignment to either the intervention or control group. Each participant selected one of the sealed and shuffled papers containing group information, ensuring that the group assignment was unpredictable and uninfluenced by the researcher. However, due to the nature of the intervention, neither the participants nor the physiotherapist were blinded to the group assignment.

### 2.6. Participant Flow and Attrition

**Figure 1:**
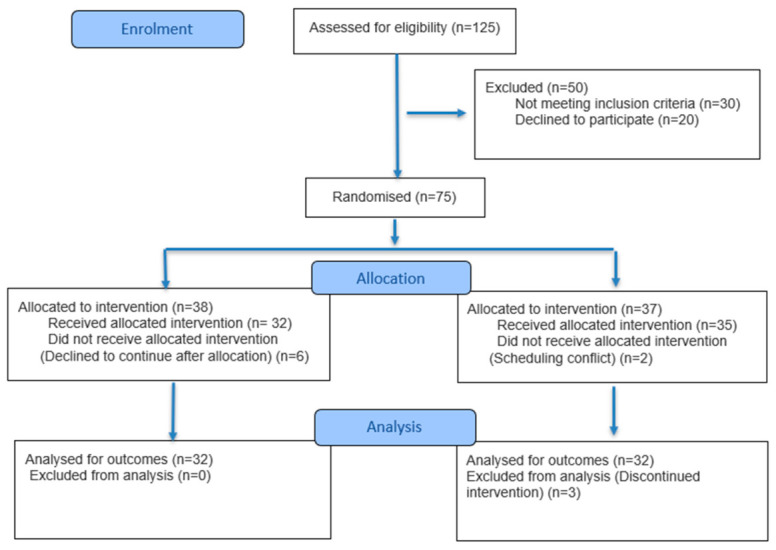
CONSORT 2025 Flow Diagram [22].

A total of 75 participants were randomised, of whom 64 (32 in each group) completed the intervention and were included in the final analyses (Figure 1). In the MBY group, six participants withdrew from the study before starting the intervention, stating that they were dissatisfied with their group assignment. In the control group, two participants withdrew due to scheduling issues, while three participants withdrew because they were unable to maintain the recommended walking programme regularly. Participants who were unable to attend MBY sessions were provided with make-up sessions at appropriate times for that week. Statistical analyses were conducted according to a per-protocol approach, including participants who completed the planned sessions. Despite participant loss, the analysed sample size met the pre-specified target number, and the statistical power of the study was adequately maintained.

### 2.7. Interventions

#### 2.7.1. Patient Education

Although the primary aim of the study was not to evaluate the effectiveness of patient education, structured patient education was provided to all participants in line with EULAR’s recommendation of patient education and non-pharmacological strategies as the initial approach to fibromyalgia management [6]. The EULAR [6] guidelines were used as a basis when preparing the education content. In addition, patient information materials from internationally reputable health organisations (Centres for Disease Control and Prevention [23], National Institute of Arthritis and Musculoskeletal and Skin Diseases [24], American College of Rheumatology [25], National Health Service [26]) were utilised. During the training, the biological basis of fibromyalgia, the heterogeneous nature of symptoms, and the necessity for multidisciplinary and individualised management were emphasised, and lifestyle strategies such as regular physical activity, sleep hygiene, activity–rest balance, and emotional health support were discussed. Furthermore, cognitive symptoms, the role of exercise, and the effects of pharmacological treatments on central pain modulation were explained, with the aim of strengthening the individual’s self-management skills.

#### 2.7.2. Control Group

The control group, consisting of individuals diagnosed with fibromyalgia, underwent a routine physiotherapy programme following patient education. The physiotherapy applications included hot packs, therapeutic ultrasound (1.5 W/cm^2^, 4 min) and Transcutaneous Electrical Nerve Stimulation (TENS) (100 Hz, 300 µs, 20 min, at sensory level) modalities and were performed for a total of 2 weeks (10 sessions) over 5 days per week. The applications were planned specifically for the neck, shoulder girdle, back, and lumbar regions, taking into account the participants’ pain and sensitivity distribution. In addition, participants were recommended a walking programme three days a week, every other day. The walking programme was organised to last 20 min (5 min warm-up, 10 min moderate-intensity main walk–based on the talk test, 5 min cool-down) and aimed to support physical activity levels. Participants continued with their medical treatments as prescribed by their physician throughout the study period. Regular phone calls were made and supportive messages were sent to participants in the control group to support continuation of the program and adherence to the walking exercises.

#### 2.7.3. Mindfulness Based Yoga Group

MBY was administered to participants in the intervention group in a group format over an 8-week period. Participants continued with the medical treatments prescribed by their physician throughout the study period, in the same manner as the control group. The intervention was conducted in six groups of five participants and two groups of six participants. Once a week, a 75-min guided group session was conducted online by a specialist physiotherapist. Online sessions were conducted via video call using the WhatsApp and WhatsApp Web platforms to facilitate participant access. This platform was chosen because it is widely and conveniently used by participants, does not require an additional link or complex login steps, is free of charge, and has no time restrictions on video calls. This ensured the sustainability of session participation and technical accessibility. On the other two days of the week, individual home exercises lasting approximately 45 min were carried out with audio recordings and video content prepared by the physiotherapist. Regular telephone calls were made to participants and supportive messages were sent to support the continuity of the home exercises.

The first week of the programme was planned as an information session. The basic concepts, attitudes, application mechanism, and safe application principles of MBY were conveyed, and body scan meditation was practised. In the second week, feedback was received from participants regarding their home practice, and body awareness exercises were conducted to support the mindfulness attitudes to be used during yoga. MBY practice began in the third week and the programme continued until the eighth week. Participant sharing was included at the beginning and end of the sessions, and suggestions were offered to support home practice. The MBY sessions were standardized using a pre-structured protocol delivered by the same physiotherapist. The structured flow and theoretical components of the MBY sessions are presented in Table 1. The programme was designed based on the mechanisms of attention regulation, body awareness, and autonomic nervous system modulation. All postures used during the practice were structured to be gentle, safe, and low-intensity. Respect for participants’ own and other participants’ personal boundaries was particularly emphasised. Where necessary, the use of supportive materials (blocks, bolsters, blankets, etc.) and modifications were allowed so that individuals could adapt their movements to their own physical needs. During the practice, each participant was encouraged to focus on their individual experience, and it was specifically stated that the work was not a competition focused on ‘doing it best’ or performance. Participants were supported in accepting their current state without judging their physical limitations and in recognising the importance of caring for the body.

### 2.8. Outcome Measures

All outcome measures in both groups were assessed at two time points: baseline and at the end of the 8-week intervention period.

#### 2.8.1. Primary Outcome Measures

The primary outcomes of this study are the change in the FIQR total score and its subscales (functioning, overall impact, and symptom severity); all other measures were treated as secondary, exploratory outcomes. This is a measure consisting of 21 items and three subscales (functioning, overall impact, symptom severity) that assesses the impact of fibromyalgia on life. The total score ranges from 0 to 100, with higher scores indicating greater disease impact [27]. The scale has been validated and is reliable in Turkish [28].

#### 2.8.2. Secondary Outcomes

Participants’ socio-demographic characteristics and information regarding the disease were recorded using the Demographic Data Form created by the researchers. The biopsychosocial status was assessed using the Cognitive Exercise Therapy Approach-Biopsychosocial Questionnaire (BETY-BQ), which consists of 30 items and has a total score ranging from 0 to 120, with higher scores indicating a more negative biopsychosocial status [29,30]. Mindfulness level was measured using the 15-item Mindful Attention Awareness Scale (MAAS), with increasing scores indicating higher levels of awareness [31]. Kinesiophobia was assessed using the 17-item Tampa Scale of Kinesiophobia (TSK). The TSK total score ranges from 17 to 68, with higher scores indicating greater fear of movement [32]. Central sensitisation symptoms were measured using the 25-item Central Sensitisation Inventory (CSI), and the total score ranges from 0 to 100, with higher values indicating increased sensitisation levels [33]. Anxiety and depression symptoms were assessed using the 14-item Hospital Anxiety and Depression Scale (HADS) [34]. Fatigue level was determined using the 9-item Fatigue Severity Scale (FSS), where high scores indicate more severe fatigue [35]. Sleep quality was assessed using the Pittsburgh Sleep Quality Index (PSQI), with total scores ranging from 0 to 21 and high scores indicating poorer sleep quality [36]. Pain catastrophising level was measured using the 13-item Pain Catastrophising Scale (PCS), where higher scores indicate a higher level of catastrophising [37]. Pain intensity was assessed using a 10-cm visual analogue scale (VAS). Participants were asked to mark their average pain intensity over the past week on a 10-cm horizontal line, with the left endpoint defined as “0 = no pain” and the right endpoint defined as “10 = unbearable/very severe pain.” [38]. All scales have established validity and reliability in Turkish [39,40,41,42,43,44,45].

### 2.9. Statistical Analysis

In this study, all statistical analyses were performed using the IBM SPSS Statistics software package (version 24.0, IBM Corp., Armonk, NY, USA). All analyses were conducted by an independent statistician. Descriptive statistics (mean ± standard deviation and percentage values) were calculated for baseline and post-intervention measurements. A chi-square homogeneity test was applied to determine whether the groups had similar distributions of demographic characteristics at baseline. To examine the effects of the intervention, a 2 × 2 mixed design analysis of variance (mixed ANOVA) was performed to assess the main effects of time (baseline and post-intervention) and group (intervention–control) factors, as well as the interaction effect of these two factors. This analysis tested the time-dependent differentiating effect of the intervention by considering the repeated measures factor (time) and the independent groups factor (group type) within the same model. The mixed ANOVA assumptions were tested prior to analysis. The independence assumption was ensured by the groups consisting of independent individuals. The normal distribution assumption was assessed for each group and time combination using the Kolmogorov–Smirnov and Shapiro–Wilk tests; normality was accepted if *p* > 0.05 (). Furthermore, skewness and kurtosis coefficients falling within the range of −2 to +2 and Z values obtained by dividing these coefficients by their standard errors remaining within the ±2.58 limits were considered as supporting indicators of normality. Meeting at least two of these criteria was considered sufficient to satisfy the normality assumption. Variance homogeneity was checked using the Levene test, confirming that the variances between groups were similar. Since the study only had two time points, the sphericity assumption was considered satisfied. After the assumptions were met, parametric analyses were performed.

In addition to mixed ANOVA, an independent groups *t*-test was used for baseline comparisons between groups. To examine changes over time in more detail on a group basis, dependent groups *t*-tests were applied for the two groups. A one-way covariance analysis was applied to evaluate the differences between groups in the post-intervention measurements. This multi-layered analysis approach allowed for the simultaneous evaluation of time-dependent changes in the intervention and differences between groups. Effect sizes were reported as partial eta squared (η^2^_p_) for ANOVA effects and Cohen’s d for within-group changes, and 95% confidence intervals (CIs) were calculated for the group × time interaction effects (expressed as the between-group difference in pre-to-post change) to support interpretation of clinical relevance. Because multiple outcomes were analysed, the FIQR total score and its subscales were designated as the primary outcomes, and all remaining measures were treated as secondary, exploratory outcomes; no formal correction for multiple comparisons was applied, and findings are therefore interpreted with caution.

## 3. Results

### 3.1. Participant Characteristics

Chi-square homogeneity tests were applied to compare the distribution of demographic characteristics between groups (Table 2). No statistically significant differences were found between the groups in terms of marital status (χ^2^ = 3.00, *p* = 0.083) and presence of children (χ^2^ = 2.78, *p* = 0.095) (*p* > 0.05). These results showed that the groups had similar characteristics in terms of basic variables related to family structure. However, there were statistically significant differences between the groups in terms of age (χ^2^ = 15.89, *p* < 0.001), educational status (χ^2^ = 29.82, *p* < 0.001), monthly income level (χ^2^ = 7.82, *p* = 0.005), and fibromyalgia disease duration (χ^2^ = 6.16, *p* = 0.046). Although some statistical differences were found in sociodemographic variables, it was observed that these differences were concentrated at the categorical level in specific subgroups and did not constitute an extreme distribution in clinical or functional terms. Furthermore, although no significant difference was found between the groups in terms of baseline scores for the primary outcome variables, the differences observed in demographic variables do not appear to be of a nature that could directly explain the effect of the intervention. Post-intervention ANCOVA statistically controlled for baseline differences. Therefore, the observed sociodemographic differences were assessed as not being of a nature that could explain the intervention effect or affect the internal validity of the study.

### 3.2. Effects of the Intervention on Outcomes

For the FIQR total score (a primary outcome), the group × time interaction was not statistically significant (F(1, 62) = 3.30, *p* = 0.074, η^2^_p_ = 0.05) (95% CI for the between-group difference in pre-to-post change: −39.78 to 1.90), indicating that the greater improvement observed in the MBY group on the primary outcome was not statistically supported. In other words, both groups improved over time, and although the reduction was numerically larger in the MBY group (FIQR total: 84.72 → 49.19) than in the control group (101.47 → 84.88), the between-group difference in the magnitude of improvement was not statistically significant. The wide confidence interval, which included zero, indicates that this estimate was imprecise and that the study was likely underpowered to detect a between-group difference on the primary outcome. The remaining FIQR subscales (also primary outcomes) and the secondary, exploratory outcomes are reported below. The study examined group (intervention/control) × time (baseline/post-intervention) interaction effects for numerous dependent variables (Table 3). A significant group × time interaction was found for Fatigue Severity (F(1, 62) = 5.67, *p* = 0.020, η^2^_p_ = 0.08). A significant interaction effect was also found for HADS-Anxiety (F(1, 62) = 7.73, *p* = 0.007, η^2^_p_ = 0.11). The group × time interaction was significant in BETY-BQ (F(1, 62) = 4.36, *p* = 0.041, η^2^_p_ = 0.06). Similarly, a significant interaction effect was observed in the BETY-BQ/Pain dimension (F(1, 62) = 12.91, *p* < 0.001, η^2^_p_ = 0.06). The interaction was significant in FIQR–Symptom Severity (F(1, 62) = 4.31, *p* = 0.042, η^2^_p_ = 0.07). A significant interaction effect was also found for CSI (F(1, 62) = 7.38, *p* = 0.009, η^2^_p_ = 0.11). Furthermore, a significant group × time interaction was found for the PCS–Rumination dimension (F(1, 62) = 6.14, *p* = 0.016, η^2^_p_ = 0.09). The 95% CIs for the between-group difference in pre-to-post change confirmed that these interaction effects excluded zero (Fatigue Severity: −15.80 to −1.38; HADS-Anxiety: −4.57 to −0.75; BETY-BQ Total: −19.32 to −0.42; BETY-BQ/Pain: −2.85 to −0.81; FIQR–Symptom Severity: −20.39 to −0.39; CSI: −17.15 to −2.61; PCS–Rumination: −5.15 to −0.55), with the negative values favouring the MBY group. A significant group × time interaction indicates only that the pattern of change from baseline to post-intervention differed between the groups; the direction of these differences was examined through the follow-up within- and between-group comparisons reported in Table 4. For the PCS-Total score, the group × time interaction was found to be marginally significant (F(1, 62) = 4.01, *p* = 0.050, partial η^2^ = 0.06). While the main effect of time was significant (F(1, 62) = 22.08, *p* < 0.001, partial η^2^ = 0.26), the main effect of group was not significant (*p* = 0.863). A general decrease in PCS scores over time was observed, and the intervention effect may have been more pronounced in the MBY group. However, group × time interactions were not statistically significant for HAD-Depression, PSQI, mindfulness, FIQR–Function, FIQR–Overall Impact, FIQR–Total, TSK, PCS–Helplessness, PCS–Magnification, and Pain intensity (VAS) variables (*p* > 0.05).

Table 3 and Table 4 address different questions and may therefore appear discrepant. The mixed-model group × time interaction (Table 3) tests whether the raw change from baseline to post-intervention differed between groups, whereas the post-intervention between-group comparison (Table 4) is adjusted for baseline values (ANCOVA). Because the groups were not fully balanced at baseline—most notably on FIQR–Function, where the baseline difference was statistically significant (*t*(62) = 2.16, *p* = 0.034), with the MBY group generally scoring better at baseline—these two models can yield different results for the same outcome, a pattern consistent with Lord’s paradox. In addition, several outcomes improved significantly within both groups (Table 4), which is consistent with the structured patient education delivered to both arms and with regression to the mean; significant within-group change therefore does not by itself indicate a treatment effect. Readers should accordingly regard the group × time interaction (Table 3) as the predefined confirmatory test and the baseline-adjusted comparisons (Table 4) as supportive. Given the modest sample size (n = 32 per group), non-significant interactions should be interpreted as inconclusive findings rather than as evidence of no effect.

The results of the within-group and between-group comparisons of the baseline and post-intervention means for the groups are presented in Table 4.

In terms of Fatigue Severity, a significant and strong decrease was observed in the MBY group after the intervention (*t*(31) = 4.77, *p* < 0.001, d = 0.86), while no significant change was found in the control group. The difference between the groups post-intervention was also statistically significant (F(1, 61) = 5.41, *p* = 0.023, η^2^ = 0.08). A significant decrease was observed in the MBY group in HADS-Anxiety symptoms (*t*(31) = 4.26, *p* < 0.001, d = 0.69), while no significant change was observed in the control group. The difference between the groups at the final measurement was significant (F(1, 61) = 5.05, *p* = 0.028, η^2^ = 0.07). BETY-BQ and BETY-BQ/Pain dimensions showed significant improvements in the MBY group, reaching large effect sizes (d = 0.84 and d = 0.83), with significant differences between groups post-intervention (*p* = 0.005 and *p* = 0.010, respectively). Significant decreases were observed in the MBY group in the Y FIQR–Overall Impact, FIQR–Symptom Severity, and FIQR-Total scores; the difference between the groups was significant in the final test (all *p* < 0.05). Significant decreases were observed in the TSK and CSI variables in the MBY group, and a significant difference in favour of the MBY group was found in the post-intervention comparisons (*p* = 0.020 and *p* = 0.010). A significant decrease was observed in the VAS variable in both groups, and a significant difference was determined between the groups in the final test (F(1, 61) = 4.33, *p* = 0.042).

Although there were significant intra-group improvements in the MBY group in HADS-Depression, PSQI, mindfulness, PCS–Helplessness, PCS–Magnification, AFÖ-PCS–Rumination and PCS–Total scores, the difference between the groups in the final test did not reach a statistically significant level (all *p* > 0.05).

## 4. Discussion

This RCT demonstrated the effectiveness of MBY training compared with conventional treatment in individuals with FM. The MBY intervention resulted in more pronounced improvements than the control group in fatigue, anxiety, pain catastrophizing, PCS–rumination, symptoms associated with central sensitization, biopsychosocial status, FIQR symptom severity, and BETY–BQ pain (cognitive dimension of pain). In contrast, although no significant between-group differences were observed in depression, sleep quality, mindfulness, functional status, kinesiophobia, and pain intensity, positive improvements were observed within the MBY group. When the FIQR subscales, which were analysed as primary outcomes alongside the total score, were evaluated, significant within-group improvements were observed across all subscales in the MBY group, with the greatest benefit relative to the control group observed for symptom severity. However, except for the symptom severity subscale, the group × time interaction did not reach statistical significance for the primary outcomes (Table 3). Although both groups improved over time, the greater post-intervention differences in favour of the MBY group for the primary outcomes, except for symptom severity, were not supported by statistically significant interactions. Therefore, these findings should be interpreted with caution in light of the study’s statistical power. The between-group advantage of MBY was more consistent for symptom severity, fatigue, anxiety, central sensitisation-related symptoms, biopsychosocial status, and pain rumination, for which the interaction analyses (Table 3) and the post-intervention between-group comparisons (Table 4) yielded concordant findings.

These findings are consistent with the literature. In a pilot RCT conducted by Carson et al. in women with FM, significant improvements in FIQR scores and functionality were reported following MBY intervention [12]. In a study containing follow-up data from the same study, it was reported that the total FIQR score remained 21.9% lower at the three-month follow-up compared to baseline and that there was a positive relationship between home-based yoga practice and treatment outcomes [46].

The results obtained in terms of fatigue levels are also consistent with previous studies reporting that yoga and mindfulness-based interventions can have positive effects on fatigue in fibromyalgia and other chronic pain conditions [11,47,48,49]. Furthermore, a meta-analysis examining non-pharmacological interventions in fibromyalgia reported that mind–body-based exercises were more effective on fatigue than aerobic exercises [50]. This can be explained by the fact that fatigue in fibromyalgia is a multidimensional symptom involving not only physical but also cognitive and emotional components. MBY may have created a regulatory effect on fatigue perception by integrating physical activity with mindfulness, breathing, and meditation components [51].

When assessed in terms of psychological symptoms, the improvement observed in anxiety levels was found to be consistent with the literature [11,52,53]. A literature review examining the effectiveness of mindfulness-based interventions in various chronic pain conditions reported that these interventions yielded positive treatment outcomes for anxiety symptoms, which frequently accompany chronic pain [54]. A meta-analysis including non-clinical populations also reported that mindful exercises were more effective than non-mindful exercises in reducing anxiety symptoms, and that yoga practices in particular provided a moderately significant effect. Therefore, it has been suggested that yoga could be used as an intervention in primary healthcare for anxiety management [55]. This positive effect on anxiety can be explained by its close relationship with autonomic nervous system activation and stress response, and by the fact that MBY incorporates the components of breathing, mindfulness, and relaxation [52].

Improvement in depression was observed in the MBY group. However, no difference emerged between the groups. This situation may be related to the presence of active interventions in the control group, such as patient education and regular walking, which are known to have positive effects on depression [56,57]. While it has been reported that mindfulness-based interventions can reduce depression, there is also a study that did not find any significant change [58]. It is thought that differences in programme components, such as intervention content, home practice, and group interaction, may influence these results.

Sleep quality is also considered an important clinical outcome area in fibromyalgia. It is thought that mindfulness-based approaches may contribute to improved sleep quality by regulating autonomic nervous system activity and reducing the stress response, thereby fostering non-judgmental acceptance of experiences and encouraging the formation of new metacognitive resources [59,60]. In our study, positive changes in sleep quality were observed in the MBY group, and these findings are consistent with studies reporting the positive effects of yoga on sleep quality and psychological symptoms in rheumatic diseases [61,62,63]. The positive effects of interventions such as patient education and aerobic exercise applied in the control group may explain the lack of difference between the groups [64,65].

In chronic pain conditions, pain, fatigue, physical inactivity, and kinesiophobia can form a vicious cycle that exacerbates the clinical picture [15]. The improvement in psychological and clinical symptoms observed in our study is likely to have contributed to a reduction in kinesiophobia. The literature reports that pain education, cognitive behavioural approaches, and exercise interventions can reduce pain-related kinesiophobia in individuals with fibromyalgia [8,66,67]. In MBY, the individual’s approach to bodily sensations with non-judgmental awareness, the application of low-intensity and safe movements, and the emphasis on an acceptance-based approach may have contributed to the reduction in kinesiophobia by decreasing the perception of movement as threatening. Furthermore, mindfulness levels have been reported to be negatively associated with some components of the fear-avoidance model [68].

Exercise intensity may also have partially influenced the findings, as MBY was planned as a low-intensity mind–body intervention, whereas walking in the active control group was planned as moderate-intensity physical activity, as recommended in the literature. However, despite the higher planned intensity of walking, some secondary outcomes favored the MBY group. Therefore, these differences may not be explained solely by exercise intensity; the biopsychosocial nature of MBY, including breath awareness, body awareness, relaxation, and mindfulness-based psychosocial components, may also have contributed to the observed improvements [6,11,12].

In terms of pain catastrophising, improvement was observed in all subscales in the MBY group, with superiority noted in the rumination subscale compared to the control group. These findings are consistent with previous studies reporting that yoga and mindfulness-based interventions can reduce pain catastrophising [12,62]. Mindfulness-based approaches may contribute to individuals re-evaluating their thoughts about pain and developing functional coping strategies [69]. Furthermore, the social support, experience sharing, and cognitive reframing processes provided by MBY and patient education conducted in a group format may also have contributed to the reduction in catastrophising tendencies. In this regard, it is thought that yoga-based multi-component interventions may help reduce maladaptive thoughts and behaviours related to pain in fibromyalgia by targeting both physical and cognitive processes. When examining mindfulness levels in relation to these cognitive processes, improvements in mindfulness and some psychological variables were reported in a pilot study conducted with women with fibromyalgia after an eight-week Hatha yoga programme [58]. However, although some studies conducted in individuals with chronic pain have observed an upward trend in mindfulness levels, it has also been reported that these changes are not statistically significant due to the trait-based nature of the scales used [70,71]. Furthermore, it has been suggested that high scores on some subscales of mindfulness scales in individuals diagnosed with fibromyalgia may be related to increased attention to bodily sensations and sensory hypervigilance rather than regulated awareness [72]. In the present study, an increase in mindfulness levels was observed within the MBY group, but no superiority was found compared to the control group. This situation may be related to factors such as the tendential structure of the scale used, the active interventions applied in the control group, and sensory amplification seen in fibromyalgia.

Pain is one of the central symptoms of fibromyalgia and is considered one of the primary outcome variables in most studies examining mind–body exercises. In our study, although a significant decrease in pain levels assessed by VAS was observed within the MBY group, this decrease was not superior to that of the control group. It is thought that physical therapy modalities applied in the control group, such as TENS, hot packs, and ultrasound, may have provided similar improvements by exhibiting a short-term peripheral analgesic effect. In contrast, the fact that BETY-BQ showed a significant superiority in the pain dimension of the MBY group compared to the control group suggests that MBY may affect not only pain intensity but also the functional and psychosocial dimensions of pain. This finding is consistent with previous studies showing that mindfulness-based approaches affect pain-related distress rather than pain perception [68,73]. Mindfulness practices may reduce reactivity to pain and the experience of suffering by separating the sensory component of pain from its cognitive and emotional components [74]. This process is associated with cortico-thalamic-cortical networks and regulatory mechanisms in related brain regions involved in the cognitive and emotional appraisal of pain [48,75,76]. Furthermore, yoga practices may contribute to pain improvement by regulating the Hypothalamic–Pituitary–Adrenal axis, supporting autonomic nervous system balance, and reducing inflammation-related biological markers [68,77,78,79].

Central sensitisation is recognised as one of the fundamental pathophysiological mechanisms of fibromyalgia and manifests clinically in patients as widespread pain sensitivity, hyperalgesia, and allodynia [80,81]. In our study, a statistically significant improvement in CSI scores was observed in favour of MBY. These results are consistent with the literature. In a telerehabilitation-based Hatha yoga study, the yoga group reported significant improvements in pain perception, central sensitisation, and sleep quality compared to the passive control group [15]. Similarly, studies investigating the effects of yoga on pain processing mechanisms have shown that it can increase the pressure pain threshold, reduce pain catastrophising, and modulate hyperalgesia [82]. In a pilot study evaluating MBY intervention using multimodal quantitative sensory testing, significant improvements were found in heat pain tolerance, pressure pain threshold, and post-pain sensations, suggesting that pain processing abnormalities specific to fibromyalgia may be alleviated [13]. However, some studies have not shown significant changes in objective pain measurements despite a decrease in perceived sensitivity; this may be related to differences in the duration, frequency, and measurement methods used in yoga interventions [12].

One of the notable aspects of our study is the implementation of the guided component of MBY via telerehabilitation. Telerehabilitation is gaining increasing importance as an effective approach that enhances access to treatment and the sustainability of interventions, particularly in chronic conditions such as fibromyalgia that require long-term management [15]. The fact that no safety issues or intervention-related dropouts were observed during the intervention process in our study further supports the view that the MBY programme delivered via telerehabilitation is a feasible and safe approach. Fibromyalgia is a multidimensional disease and is currently recommended to be addressed within the biopsychosocial model framework [83]. Our study showed that BETY-BQ results demonstrated a more pronounced improvement in the MBY group compared to the control group. The BETY-BQ’s assessment of multidimensional areas such as pain level, daily living activities, emotional state, and social interaction indicates that MBY can positively affect not only pain but also patients’ overall functioning and psychosocial well-being. These findings are consistent with previous studies reporting that mindfulness-based interventions can improve fibromyalgia symptoms through neurobiological, psychological, and behavioural mechanisms [11,70,77,84]. Carson et al. also demonstrated that MBY intervention produced significant improvements in both clinical symptoms and psychosocial domains, and that these effects were sustainable during follow-up [12,45]. These findings support the notion that MBY may be an effective complementary rehabilitation approach that holistically targets the biopsychosocial dimensions of fibromyalgia.

## 5. Limitations and Strengths

This study has certain limitations. Firstly, the fact that the sample consisted solely of female participants may limit the generalisability of the findings, although it is consistent with the literature. Some post-randomization withdrawals were observed in our study. It has been reported that, in behavioral and rehabilitation-based interventions, such withdrawals may be related to participants’ treatment preferences, expectations, or familiarity with the allocated intervention, and may occur in this type of study. The follow-up of home exercises was also based on participants’ self-reports. Furthermore, due to the nature of the intervention, participants and physiotherapists could not be blinded; therefore, placebo effects and expectancy-related bias cannot be completely ruled out. The lack of long-term follow-up assessments and the absence of assessor blinding are other limitations of the study. In addition, because a large number of secondary outcomes were analysed without a formal correction for multiple comparisons, the secondary findings should be regarded as exploratory and interpreted with caution given the increased risk of Type I error.

This study has several strengths. When the findings are evaluated together, it is thought that this study may contribute to the literature in terms of both clinical outcomes and intervention design. This study contributes to the limited literature on telerehabilitation-based interventions by evaluating mindfulness-based yoga training delivered via telerehabilitation in individuals diagnosed with fibromyalgia from a biopsychosocial perspective within an active-controlled randomized design. Furthermore, the use of an active care model in the comparison group, which included patient education, walking, and physical therapy modalities, is one of the original aspects of this study. No safety issues or unexpected side effects were observed during the intervention process, and there was no intervention-related loss after the MBY programme began.

## 6. Conclusions

The findings of this RCT suggest that the MBY intervention was associated with more favorable short-term results than the control group in several secondary outcomes, including fatigue, anxiety, symptoms associated with central sensitization, biopsychosocial status, the cognitive dimension of pain, FIQR symptom severity, pain catastrophizing, and pain-related ruminative thoughts. These findings are consistent with the multidimensional nature of fibromyalgia and suggest that MBY applications may be a promising complementary approach to reducing symptom burden. Furthermore, the delivery of the intervention via telerehabilitation suggests a potential approach for accessible and sustainable intervention models in fibromyalgia management.

Future studies with higher methodological quality, larger and more heterogeneous samples, long-term follow-up assessments, intention-to-treat analyses, and physiological/neurophysiological measurement methods may provide a more comprehensive and reliable understanding of the effects of these interventions. Evaluating the cost-effectiveness of MBY interventions delivered via telerehabilitation may also contribute to a better understanding of the feasibility of these approaches in clinical practice.

## Figures and Tables

**Table 1:** MBY Protocol Structured Session Flow.

Stage	Duration	Application Content	Mindfulness Attitude	Clinical Purpose
Introduction	5 min	Breath awareness, observing your current emotional and physical state (checking in), setting intentions	Present moment orientation, non-judgmental awareness	Attention regulation, initial focus
Warm-up	5–10 min	Gentle, somatic and mindful warm-up movements (1–3 basic movements/postures)	Body awareness, self-compassion, discovering your boundaries, protecting them	Safe movement preparation, proprioceptive activation
Positions	5–10 min	Alignment-focused, low-intensity mindful yoga poses	Acceptance, patience, mind–body integrity	Postural awareness, motor control
Cool-down	5–10 min	Yin or restorative yoga poses (1–2 positions)	Letting go, relaxation	Parasympathetic activation
Relaxation	5–10 min	Deep breathing, body scan or guided relaxation	Inner observation, reduced reactivity	Autonomic regulation, stress reduction
Completion	5–10 min	Remembering one’s intention and internal orientation, closure	Internal integration, viewing experience holistically	Integration of experience

**Table 2:** Participants’ identifying characteristics.

Characteristics	Subgroup	MBY		Control	
		n	%	n	%
**Age**	35 years old and under	13	40.60	2	6.20
	36–45 years old	10	31.20	6	18.80
	46 years old and over	9	28.10	5	75.00
		*x*^2^ = 15.89		*p* < 0.001	
**Educational Status**	Secondary school and below	1	3.10	19	59.40
	High school	6	18.80	8	25.00
	Undergraduate and above	25	78.10	5	15.60
		*x*^2^ = 29.82		*p* < 0.001	
**Marital Status**	Single	11	34.40	5	15.60
	Married	21	65.60	27	84.40
		*x*^2^ = 3.00		*p* = 0.083	
**Presence of Children**	Yes	20	62.50	26	81.20
	No	12	37.50	6	18.80
		*x*^2^ = 2.78		*p* = 0.095	
**Monthly income**	Low	2	6.20	11	34.40
	Medium–High	30	93.80	21	65.60
		*x*^2^ = 7.82		*p* = 0.005	
**Duration of Fibromyalgia**	0–5 year	18	62.10	16	57.10
	6–10 year	3	1.30	10	35.70
	11 year and older	8	27.60	2	7.10
		*x*^2^ = 6.16		*p* = 0.046	

The chi-square homogeneity test was used in the statistical analyses. n: number of participants; %: percentage; χ^2^: chi-square test statistic; *p*: level of statistical significance.

**Table 3:** Mixed measures variance analysis results for the subscale and total scale scores of participants in the MBY and control groups.

Factor	Source	Sum of Squares	df	Mean Square	F	*p*	Partial η^2^
**FIQR–Function**	Between groups	62,515.28	63				
	Groups (MBY-C)	7761.04	1	7761.04	8.93	0.004	0.12
	Error	54,754.24	62	869.12			
	Within groups	18,404.77	64				
	Time (Measurement)	1369.80	1	1369.80	5.23	0.026	0.08
	Group × Time	529.89	1	529.89	2.02	0.16	0.03
	Error	16,505.08	62	261.99			
**FIQR–Overall Impact**	Between groups	3641.51	63				
	Groups (MBY-C)	190.13	1	190.13	3.42	0.069	-
	Error	3451.38	62	55.68			
	Within groups	2274.01	64				
	Time (Measurement)	338.00	1	338.00	10.86	0.002	0.15
	Group × Time	6.13	1	6.13	0.20	0.659	-
	Error	1929.88	62	31.13			
**FIQR–Symptom Severity**	Between groups	7140.72	63				
	Groups (MBY-C)	2363.28	1	2363.28	3.07	0.085	-
	Error	4777.44	62	770.60			
	Within groups	21,470.01	64				
	Time (Measurement)	8320.50	1	8320.50	41.68	<0.001	0.4
	Group × Time	861.13	1	861.13	4.31	0.042	0.07
	Error	12,378.38	62	199.65			
**FIQR–Total**	Between groups	226,372.50	63				
	Groups (MBY-C)	21,997.53	1	21,997.53	6.67	0.012	0.1
	Error	204,374.97	62	3296.37			
	Within groups	78,487	64				
	Time (Measurement)	21,736.13	1	21,736.13	25.01	<0.001	0.29
	Group × Time	2869.03	1	2869.03	3.30	0.074	0.05
	Error	53,881.84	62	869.06			
**BETY–BQ Total**	Between groups	58,652.22	63				
	Groups (MBY-C)	4209.03	1	4209.03	4.79	0.032	0.07
	Error	54,443.19	62	878.12			
	Within groups	18,041.01	64				
	Time (Measurement)	6160.50	1	6160.50	34.41	<0.001	0.36
	Group × Time	780.13	1	780.13	4.36	0.041	0.06
	Error	11,100.38	62	179.04			
**BETY–BQ Pain**	Between groups	1712.68	63				
	Groups (MBY-C)	159.76	1	159.76	6.38	0.014	0.09
	Error	1552.92	62	25.05			
	Within groups	765.51	64				
	Time (Measurement)	159.76	1	159.76	17.12	<0.001	0.22
	Group × Time	120.45	1	120.45	12.91	<0.001	0.06
	Error	578.55	62	9.33			
**Mindfulness**	Between groups	26,147.47	63				
	Groups (MBY-C)	42.78	1	42.78	0.10	0.751	-
	Error	26,104.69	62	421.04			
	Within groups	5618	64				
	Time (Measurement)	520.03	1	520.03	6.35	0.014	0.09
	Group × Time	22.78	1	22.78	0.29	0.60	-
	Error	5075.19	62	81.86			
**Fatigue Severity**	Between groups	20,602.25	63				
	Groups (MBY-C)	303.20	1	303.20	0.93	0.34	
	Error	20,299.05	62	327.40			
	Within groups	8623.50	64				
	Time (Measurement)	1575.01	1	1575.01	15.12	<0.001	0.20
	Group × Time	590.82	1	590.82	5.67	0.02	0.08
	Error	6457.67	62	104.16			
**HADS–Anxiety**	Between groups	2071.93	63				
	Groups (MBY-C)	10.70	1	10.70	0.32	0.573	-
	Error	2061.23	62	33.25			
	Within groups	576.50	64				
	Time (Measurement)	67.57	1	67.57	9.26	0.003	0.13
	Group × Time	56.45	1	56.45	7.73	0.007	0.11
	Error	452.48	62	7.30			
**HADS–Depression**	Between groups	1762.93	63				
	Groups (MBY-C)	61.88	1	61.88	2.26	0.138	-
	Error	1701.05	62	27.44			
	Within groups	391.50	64				
	Time (Measurement)	29.07	1	29.07	5.14	0.027	0.08
	Group × Time	11.88	1	11.88	2.10	0.152	-
	Error	350.55	62	5.65			
**PSQI**	Between groups	660.74	63				
	Groups (MBY-C)	1.32	1	1.32	0.124	0.726	-
	Error	659.42	62	10.64			
	Within groups	318.51	64				
	Time (Measurement)	37.20	1	37.20	8.26	0.006	0.12
	Group × Time	2.26	1	2.26	0.50	0.481	-
	Error	279.05	62	4.50			
**TSK**	Between groups	5415.99	63				
	Groups (MBY-C)	223.13	1	223.13	2.66	0.108	-
	Error	5192.86	62	83.76			
	Within groups	1516.52	64				
	Time (Measurement)	354.46	1	354.46	19.88	<0.001	0.24
	Group × Time	56.45	1	56.45	3.17	0.080	-
	Error	1105.61	62	17.83			
**CSI**	Between groups	28,601.50	63				
	Groups (MBY-C)	87.78	1	87.78	0.19	0.664	-
	Error	28,513.72	62	459.90			
	Within groups	10,518	64				
	Time (Measurement)	3180.03	1	3180.03	30.07	<0.001	0.33
	Group × Time	780.13	1	780.13	7.38	0.009	0.11
	Error	6557.84	62	105.77			
**PCS–Helplessness**	Between groups	2840.36	63				
	Groups (MBY-C)	13.13	1	13.13	0.29	0.593	-
	Error	2827.23	62	45.60			
	Within groups	1487.50	64				
	Time (Measurement)	361.13	1	361.13	21.15	<0.001	0.25
	Group × Time	67.57	1	67.57	3.96	0.051	-
	Error	1058.80	62	17.08			
**PCS–Magnification**	Between groups	944.88	63				
	Groups (MBY-C)	4.50	1	4.50	0.30	0.588	-
	Error	940.38	62	15.17			
	Within groups	553.01	64				
	Time (Measurement)	105.13	1	105.13	14.70	<0.001	0.19
	Group × Time	4.50	1	4.50	0.63	0.431	-
	Error	443.38	62	7.15			
**PCS–Rumination**	Between groups	2165.80	63				
	Groups (MBY-C)	9.57	1	9.57	0.28	0.602	-
	Error	2156.23	62	34.78			
	Within groups	910.50	64				
	Time (Measurement)	192.57	1	192.57	18.28	<0.001	0.23
	Group × Time	64.70	1	64.70	6.14	0.016	0.09
	Error	653.23	62	10.54			
**PCS Total**	Between groups	14,613.47	63				
	Groups (MBY-C)	7.03	1	7.03	0.03	0.863	0.01
	Error	14,606.44	62	235.59			
	Within groups	7422.00	64				
	Time (Measurement)	1860.50	1	1860.50	22.08	<0.001	0.26
	Group × Time	338.00	1	338.00	4.01	0.050	0.06
	Error	5223.50	62	84.25			
**VAS**	Between groups	1017.31	63				
	Groups (MBY-C)	96.26	1	96.26	6.48	0.013	0.10
	Error	921.05	62	14.86			
	Within groups	413.50	64				
	Time (Measurement)	106.95	1	106.95	25.34	<0.001	0.29
	Group × Time	4.88	1	4.88	1.16	0.286	-
	Error	261.67	62	4.22			

In statistical analyses, 2 × 2 mixed measures variance analysis was used. MBY: Mindfulness-Based Yoga; C: Control group; df: Degrees of freedom; F: F statistic; *p*: *p*-value; η^2^ (partial eta squared): Effect size; Group × Time: Interaction effect between group and time; Time: Measurement factor (baseline–post-intervention). The FIQR total score and its subscales were the predefined primary outcomes; all remaining variables were secondary, exploratory outcomes. Table 3 reports the primary inferential model (the group × time interaction), whereas Table 4 presents the follow-up within-group and between-group comparisons for the same outcomes.

**Table 4:** Results of within-group and between-group comparisons of baseline and post-intervention means for participants in the MBY and control groups.

Subfactor	Measurement	MBY Group		Control Group		Between-Group
		Min–Max	X^−^ ± SD	Min–Max	X^−^ ± SD	
	Baseline	0–77	26.25 ± 20.79	0–82	37.59 ± 21.18	*t*(62) = 2.16, *p* = 0.034, d = 0.54
**FIQR–Function**	Post-intervention	0–112	15.72 ± 27.02	0–82	34.75 ± 26.09	F(1, 61) = 3.68, *p* = 0.060, η^2^ = 0.03
	**Within-group**	***t*** **(31) = 2.93, *p* = 0.006, d = 0.44**		***t*** **(31) = 0.63, *p* = 0.531, d = 0.12**		
	Baseline	0–20	7.31 ± 7.16	0–20	9.31 ± 6.69	*t*(62) = 1.15, *p* = 0.253, d = 0.23
**FIQR–Overall Impact**	Post-intervention	0–20	3.63 ± 5.77	0–20	6.50 ± 6.65	F(1, 61) = 5.37, *p* = 0.024, η^2^ = 0.08
	**Within-group**	***t*** **(31) = 2.93, *p* = 0.006, d = 0.57**		***t*** **(31) = 1.85, *p* = 0.073, d = 0.31**		
	Baseline	0–100	51.16 ± 25.95	10–90	54.56 ± 18.46	*t*(62) = 0.61, *p* = 0.547, d = 0.15
**FIQR–Symptom Severity**	Post-intervention	0–84	29.84 ± 23.84	14–76	43.63 ± 18.92	F(1, 61) = 7.28, *p* = 0.009, η^2^ = 0.11
	**Within-group**	***t*** **(31) = 5.39, *p* < 0.001, d = 0.86**		***t*** **(31) = 3.58, *p* = 0.001, d = 0.65**		
	Baseline	0–186	84.72 ± 48.55	19–187	101.47 ± 38.10	*t*(62) = 1.54, *p* = 0.130, d = 0.38
**FIQR-Total**	Post-intervention	0–182	49.19 ± 50.22	14–172	84.88 ± 44.72	F(1, 61) = 6.38, *p* = 0.014, η^2^ = 0.10
	**Within-group**	***t*** **(31) = 4.80, *p* < 0.001, d = 0.85**		***t*** **(31) = 2.26, *p* = 0.031, d = 0.40**		
	Baseline	0–77	26.25 ± 20.79	0–82	37.59 ± 21.18	*t*(62) = 2.16, *p* = 0.034, d = 0.54
**FIQR–Function**	Post-intervention	0–112	15.72 ± 27.02	0–82	34.75 ± 26.09	F(1, 61) = 3.68, *p* = 0.060, η^2^ = 0.03
	**Within-group**	***t*** **(31) = 2.93, *p* = 0.006, d = 0.44**		***t*** **(31) = 0.63, *p* = 0.531, d = 0.12**		
	Baseline	4–17	10.31 ± 3.63	3–20	11.63 ± 4.48	*t*(62) = 1.29, *p* = 0.203, d = 0.32
**BETY–BQ/Pain**	Post-intervention	1–18	7.16 ± 3.74	3–19	10.31 ± 4.63	F(1, 61) = 7.02, *p* = 0.010, η^2^ = 0.10
	**Within-group**	***t*** **(31) = 4.72, *p* < 0.001, d = 0.83**		***t*** **(31) = 1.54, *p* = 0.132, d = 0.27**		
	Baseline	24–83	53.63 ± 16.21	18–85	55.63 ± 18.32	*t*(62) = 0.46, *p* = 0.645, d = 0.12
**Mindfulness**	Post-intervention	18–78	58.50 ± 14.23	28–85	58.81 ± 14.32	F(1, 61) = 0.10, *p* = 0.760, η^2^ = 0.01
	**Within-group**	***t*** **(31) = 3.20, *p* = 0.003, d = 0.32**		***t*** **(31) = 1.13, *p* = 0.266, d = 0.18**		
	Baseline	17–63	46.75 ± 12.10	22–63	45.53 ± 11.97	*t*(62) = 0.41, *p* = 0.687, d = 0.10
**Fatigue Severity**	Post-intervention	12–63	35.44 ± 14.24	14–122	42.81 ± 19.25	F(1, 61) = 5.41, *p* = 0.023, η^2^ = 0.08
	**Within-group**	***t*** **(31) = 4.77, *p* < 0.001, d = 0.86**		***t*** **(31) = 1.00, *p* = 0.325, d = 0.16**		
	Baseline	1–19	10.91 ± 4.55	0–20	9.00 ± 5.24	*t*(62) = 1.56, *p* = 0.125, d = 0.32
**HADS–Anxiety**	Post-intervention	1–14	8.13 ± 3.47	0–19	8.88 ± 4.58	F(1, 61) = 5.05, *p* = 0.028, η^2^ = 0.07
	**Within-group**	***t*** **(31) = 4.26, *p* < 0.001, d = 0.69**		***t*** **(31) = 0.18, *p* = 0.859, d = 0.03**		
	Baseline	0–17	7.31 ± 4.11	1–20	8.09 ± 3.75	*t*(62) = 0.79, *p* = 0.430, d = 0.20
**HADS–Depression**	Post-intervention	0–16	5.75 ± 4.33	0–19	7.75 ± 4.06	F(1, 61) = 3.29, *p* = 0.074, η^2^ = 0.03
	**Within-group**	***t*** **(31) = 2.38, *p* = 0.024, d = 0.37**		***t*** **(31) = 0.66, *p* = 0.517, d = 0.09**		
	Baseline	1–15	6.69 ± 3.20	3–13	6.63 ± 2.61	*t*(62) = 0.09, *p* = 0.932, d = 0.02
**PSQI**	Post-intervention	2–13	5.34 ± 2.82	2–12	5.81 ± 2.29	F(1, 61) = 0.69, *p* = 0.410, η^2^ = 0.01
	**Within-group**	***t*** **(31) = 2.54, *p* = 0.016, d = 0.45**		***t*** **(31) = 1.53, *p* = 0.137, d = 0.28**		
	Baseline	23–60	39.75 ± 6.59	24–55	41.06 ± 8.00	*t*(62) = 0.72, *p* = 0.477, d = 0.18
**TSK**	Post-intervention	23–56	35.09 ± 6.61	23–48	39.06 ± 7.21	F(1, 61) = 5.66, *p* = 0.020, η^2^ = 0.09
	**Within-group**	***t*** **(31) = 4.52, *p* < 0.001, d = 0.71**		***t*** **(31) = 1.85, *p* = 0.074, d = 0.25**		
	Baseline	21–100	52.44 ± 17.76	20–91	49.16 ± 17.15	*t*(62) = 0.75, *p* = 0.455, d = 0.19
**CSI**	Post-intervention	8–86	37.53 ± 17.08	16–75	44.13 ± 15.16	F(1, 61) = 4.33, *p* = 0.010, η^2^ = 0.11
	**Within-group**	***t*** **(31) = 6.03, *p* < 0.001, d = 0.86**		***t*** **(31) = 1.88, *p* = 0.069, d = 0.29**		
	Baseline	2–24	13.41 ± 5.28	0–24	11.31 ± 6.31	*t*(62) = 1.44, *p* = 0.155, d = 0.32
**PCS–Helplessness**	Post-intervention	0–21	8.59 ± 5.25	1–24	9.41 ± 5.48	F(1, 61) = 1.92, *p* = 0.171, η^2^ = 0.02
	**Within-group**	***t*** **(31) = 5.94, *p* < 0.001, d = 0.91**		***t*** **(31) = 1.57, *p* = 0.127, d = 0.32**		
	Baseline	0–12	6.25 ± 3.29	0–12	5.50 ± 3.71	*t*(62) = 0.86, *p* = 0.395, d = 0.21
**PCS–Magnification**	Post-intervention	0–11	4.06 ± 3.23	0–11	4.06 ± 3.10	F(1, 61) = 0.11, *p* = 0.745, η^2^ = 0.01
	**Within-group**	***t*** **(31) = 3.72, *p* = 0.001, d = 0.67**		***t*** **(31) = 1.94, *p* = 0.061, d = 0.40**		
	Baseline	0–16	8.38 ± 4.66	0–16	7.50 ± 5.44	*t*(62) = 0.69, *p* = 0.492, d = 0.17
**PCS–Rumination**	Post-intervention	0–16	4.50 ± 4.10	0–16	6.47 ± 4.75	F(1, 61) = 1.92, *p* = 0.171, η^2^ = 0.02
	**Within-group**	***t*** **(31) = 4.94, *p* < 0.001, d = 0.88**		***t*** **(31) = 1.23, *p* = 0.227, d = 0.19**		
	Baseline	2–52	28.03 ± 12.17	0–52	24.31 ± 14.22	*t*(62) = 1.12, *p* = 0.265, d = 0.28
**PCS–Total**	Post-intervention	0–47	17.16 ± 12.02	1–50	19.94 ± 12.07	F(1, 61) = 2.65, *p* = 0.109, η^2^ = 0.02
	**Within-group**	***t*** **(31) = 5.45, *p* < 0.001, d = 0.96**		***t*** **(31) = 1.71, *p* = 0.097, d = 0.30**		
	Baseline	0–10	5.28 ± 3.29	0–10	6.63 ± 2.87	*t*(62) = 1.74, *p* = 0.087, d = 0.36
**VAS**	Post-intervention	0–10	3.06 ± 3.17	0–10	5.19 ± 2.99	F(1, 61) = 4.33, *p* = 0.042, η^2^ = 0.07
	**Within-group**	***t*** **(31) = 3.97, *p* < 0.001, d = 0.75**		***t*** **(31) = 3.10, *p* = 0.004, d = 0.59**		

MBY = Mindfulness-Based Yoga; Min–Max = minimum–maximum values; X^−^ ± SD = mean ± standard deviation; *t* = *t*-test statistic; F = analysis of variance statistic; *p* = *p*-value; d = Cohen’s d effect size; η^2^ = partial eta squared effect size.

## Data Availability

The datasets generated and analyzed during the current study are available from the corresponding author upon reasonable request.
